# Effect of glucose depletion and fructose administration during chondrogenic commitment in human bone marrow-derived stem cells

**DOI:** 10.1186/s13287-022-03214-2

**Published:** 2022-12-27

**Authors:** Daniele Zuncheddu, Elena Della Bella, Dalila Petta, Cecilia Bärtschi, Sonja Häckel, Moritz C. Deml, Martin J. Stoddart, Sibylle Grad, Valentina Basoli

**Affiliations:** 1 AO Research Institute Davos, 7270 Davos Platz, Switzerland; 2 Regenerative Medicine Technologies Laboratory, Laboratories for Translational Research (LRT), Ente Ospedaliero Cantonale (EOC), 6500 Bellinzona, Switzerland; 3 Service of Orthopaedics and Traumatology, Department of Surgery, Ente Ospedaliero Cantonale (EOC), 6903 Lugano, Switzerland; 4 Department of Orthopaedic Surgery and Traumatology, Inselspital, Bern University Hospital, University of Bern, 3010 Bern, Switzerland

**Keywords:** Human mesenchymal stem cells, Differentiation, Chondrogenesis, Cartilage, Fructose, Glucose

## Abstract

**Background:**

Bone marrow mesenchymal stromal cells (BMSCs) are promising for therapeutic use in cartilage repair, because of their capacity to differentiate into chondrocytes. Often, in vitro differentiation protocols employ the use of high amount of glucose, which does not reflect cartilage physiology. For this reason, we investigated how different concentrations of glucose can affect the chondrogenic differentiation of BMSCs in cell culture pellets. Additionally, we investigated how fructose could influence the chondrogenic differentiation in vitro.

**Methods:**

BMSC were isolated from six donors and cultured in DMEM containing glucose at either 25 mM (HG), 5.5 mM (LG) or 1 mM (LLG), and 1% non-essential amino acids, 1% ITS+, in the presence of 100 nM dexamethasone, 50 µg/ml ascorbic acid-2 phosphate and 10 ng/ml TGF-*β*1. To investigate the effect of different metabolic substrates, other groups were exposed to additional 25 mM fructose. The media were replaced every second day until day 21 when all the pellets were harvested for further analyses. Biochemical analysis for glycosaminoglycans into pellets and released in medium was performed using the DMMB method. Expression of GLUT3 and GLUT5 was assayed by qPCR and validated using FACS analysis and immunofluorescence in monolayer cultures. Chondrogenic differentiation was further confirmed by qPCR analysis of *COL2A1*, *COL1A1*, *COL10A1*, *ACAN*, *RUNX2*, *SOX9*, *SP7*, *MMP13*, and *PPARG*, normalized on *RPLP0*. Type 2 collagen expression was subsequently validated by immunofluorescence analysis.

**Results:**

We show for the first time the presence of fructose transporter GLUT5 in BMSC and its regulation during chondrogenic commitment. Additionally, decreasing glucose concentration during chondrogenesis dramatically decreased the yield of differentiation. However, the use of fructose alone or together with low glucose concentrations does not limit cell differentiation, but on the contrary it might help in maintaining a stable chondrogenic phenotype comparable with the standard culture conditions (high glucose).

**Conclusion:**

This study provides evidence that BMSC express GLUT5 and differentially regulate GLUT3 in the presence of glucose variation. This study gives a better comprehension of BMSCs sugar use during chondrogenesis.

**Supplementary Information:**

The online version contains supplementary material available at 10.1186/s13287-022-03214-2.

## Background

The branch of regenerative medicine focusing on the therapeutic effect of mesenchymal stem cells (MSCs) for cartilage repair has gained considerable interest over the past 20 years. This is partly due to the ambition to generate tissues in vitro that could subsequently be implanted in patients (e.g. after focal cartilage injury) and partly due to advances in the development of cell therapies that can accelerate endogenous tissue healing. However, the high inter-donor variability of MSCs and of their biological responses to external stimuli probably represents one of the biggest limitations for the direct and safe translation of research results on MSCs into clinical practice [1]. In addition, effective use can only be guaranteed by the selection of the right cell source, correct cell processing [1, 2], the right amount of cells [3], everything in compliance with the minimum quality parameters that assure safety and efficacy [4]. Also, in vitro culturing, expansion and differentiation of isolated MSCs represent a potential pitfall [5]. Among many factors, protocols that do not mimic a proper physiological environment have been employed for years for cell cultures [6]. This was based on the assumption that the same media components are suitable and effective for any type of cells, regardless of tissue or donor source that might result in a different (epi)genotype and a different response to external stimuli. A crucial, but often overlooked factor, is the concentration of glucose used in expansion and differentiation media. Recently, Mantipragada and colleagues reported that the glucose concentration used for chondrogenic differentiation (25 mM in Dulbecco's Modified Eagle Medium—DMEM) is remarkably higher compared to its physiological concentrations in blood (3.9–5.6 mM normal fasting glucose concentration), synovial fluid, and cartilage tissue [7]. Cartilage is an avascular and hypoxic tissue; physiologically it shows low gas levels [8] and a glucose concentration up to 1 mM [9–11]. Glucose is the main source of energy that allows cartilage to survive and anaerobic glycolysis is the main mechanism of ATP production in chondrocytes [12]. Moreover, glucose is also needed for the production of matrix glycosaminoglycans. Glucose diffuses through the various zones resulting in a different concentration at the hypertrophic layer that requires a higher amount compared to the superficial zone [13]. For energy production, glucose metabolism initiates with the transport of the sugar into cells, which is metabolized first to glucose 6-phosphate (G6P), then to fructose 6-phospate (F6P), and the latter can enter several pathways. Most glucose will enter glycolysis to form pyruvate and either converted to lactate to produce NAD+ or transferred into the mitochondria and converted into acetyl-CoA that will enter the tricarboxylic acid cycle and oxidative phosphorylation. In addition to glycolysis and oxidative phosphorylation, some glucose can be converted to glycogen, for tissue storage, and some may enter the pentose phosphate pathway (PPP), to make nucleotides and contribute to cell proliferation [12].

Sun et al. already described how glucose concentrations regulate chondro-osteogenic differentiation of human cartilage endplate cells via *O*-linked-*N*-acetylglucosaminylation (*O*-GlcNAcylation) of SRY-box transcription factor 9 (*SOX9)* and Runt-related transcription factor 2 (*RUNX2)*; they demonstrated that a high concentration of glucose (HG: 25 mM) promoted osteogenic differentiation, whereas lower concentrations of glucose (1 and 5.5 mM)-enhanced chondrogenic differentiation [14]. Hence, an optimal concentration of glucose may exist that would provide sufficient energy for cell metabolism while inducing sustained chondrogenesis.

Fructolysis is another process for energy production starting from fructose and shares most of the same enzymes and metabolic intermediates with the glycolysis pathway. However, while glucose is metabolized directly throughout the body, fructose metabolizes predominantly in the liver [15, 16]. However, other cells than those from the liver express the fructose transporters or metabolizing enzymes, suggesting that fructose can also be used in other tissues. For example, the hexokinase present in muscle and adipose tissue phosphorylates fructose, which then enters glycolysis.

The main transporters for sugars into cells are members of the GLUT family (*SLC2Ax* gene symbol). GLUT3 (*SLC2A3*) is a high-affinity transporter for glucose, whereas GLUT5 (*SLC2A5*) is responsible for the uptake of fructose together with GLUT2. GLUT3 is also involved in the transport of mannose, galactose, and xylose but is unable to transport fructose, and it has been shown to be present in all cell types. On the contrary, GLUT5 is mainly expressed in small intestine, kidney, adipocytes, skeletal muscle, and brain but has not been described yet in hBMSCs [13, 17]. Additionally, it has been shown that three GLUT isoforms (GLUT1, GLUT3 and GLUT9) are expressed by healthy articular chondrocytes, thus influencing the chondrocyte physiology and metabolism in cartilage matrices [13].

In this work, we aim to study in vitro the effect of low glucose concentrations on the survival and chondrogenic differentiation of hBMSCs in pellet culture, focusing on the effect of fructose as an alternative energy substrate. Therefore, we investigated whether alternative metabolic pathways such as fructolysis could be involved during chondrogenesis. We hypothesized that (1) hBMSCs express GLUT5, one of the main fructose transporters, that would allow the uptake and potential use of this sugar and (2) that chondrogenesis could be sustained by fructolysis while using a low amount of glucose or even in the absence of glucose during cell commitment. This observation could pave the way to further studies about hBMSC metabolism during chondrogenic differentiation and contribute to the understanding of the role of alternative metabolites in the promotion and inhibition of cartilage homeostasis.

## Methods

### Cell isolation and culture

Bone marrow aspirates were obtained from patients undergoing spine surgery at the Inselspital Bern. The Swiss Human Research Act does not apply to research which involves anonymized biological material and/or anonymously collected or anonymized health-related data [18, 19]. General Consent which also covers anonymization of health-related data and biological material was obtained from all cell donors.

Bone marrow was aspirated from vertebral bodies of six patients (Additional file 1: Table S1) undergoing spinal surgery (4 F/2 M, age 74.3 ± 13.8 years old). hBMSCs were isolated using a standardized procedure as previously described [20]. hBMSCs were seeded at an initial cell density of 3 × 10^3^ cells/cm^2^ and grown until passage 3 (p3) in Minimum Essential Medium Eagle-alpha modification (*α*-MEM, Gibco, Thermo Fisher, Zürich, Switzerland) with the addition of 10% MSC-qualified FBS (Pan-Biotech, Aidenbach, Germany), 100 U/ml penicillin, 100 µg/ml streptomycin (Gibco) and 5 ng/ml basic fibroblast growth factor (bFGF, Fitzgerald Industries International, Acton, MA, USA). Cultures were kept in a 37 °C/5% CO_2_ humidified atmosphere and the medium was refreshed every second day until 70% confluence.

### Flow cytometry

The surface expression of glucose and fructose transporters GLUT3 and GLUT5 was analysed in naïve hBMSCs (*n* = 4 donors) by flow cytometry. Cells were detached, counted, and fixed in 4% neutral-buffered formalin (Formafix AG, Hittnau, Switzerland). Unspecific binding sites were blocked using a 2% FBS solution in PBS (hence FACS buffer). Cells were divided into different staining groups, in the number of 0.5 × 10^6^ cells each. For GLUT3 staining, cells were stained with 5 µl (1 µg/µl) of a rabbit polyclonal antibody (#PA5-99169, Thermo Fisher) for 30 min at RT, followed by incubation with 10 µg/ml of an AF488-labelled secondary goat anti-rabbit antibody (#A-11008, Thermo Fisher) for 30 min at 4 °C. For GLUT5, cells were stained with 5 µl (0.6 µg/µl) of a mouse monoclonal antibody (#TA500575, Thermo Fisher) for 30 min at RT, followed by incubation with 10 µg/ml of an AF660-labelled secondary goat anti-mouse antibody (A-21055, Thermo Fisher) for 30 min at 4 °C. Proper unstained and secondary antibody-only samples were included for determining the fluorescence positivity regions. Cells were acquired using a BD FACSAria III Cell Sorter (Becton Dickinson, Allschwil, Switzerland) and results were analysed using FlowJo™ Software for Windows v.10 (Becton Dickinson).

### Chondrogenic differentiation

Chondrogenic differentiation of hBMSCs between passages 4 and 5 was achieved in 3D pellet culture [21]. hBMSCs (2 × 10^5^ cells per pellet) were seeded in V-bottom 96-well plates (Corning) and centrifuged at 400 g for 5 min. hBMSCs were committed towards the chondrogenic phenotype by using a chondrogenic medium, composed of DMEM containing glucose at either 25 mM (HG), 5.5 mM (LG) or 1 mM (LLG), and 1% non-essential amino acids (Gibco), 1% ITS + (Corning), in the presence of 100 nM dexamethasone (Sigma-Aldrich), 50 µg/ml ascorbic acid-2 phosphate (Sigma-Aldrich) and 10 ng/ml TGF-*β*1 (Fitzgerald). To investigate the effect of different metabolic substrates, other groups of cells were exposed to additional 25 mM fructose (Sigma-Aldrich) (refer to Additional file 1: Table S2 for a complete overview of the experimental groups). The media were replaced every second day until day 21 when all the pellets were harvested for further analyses.

Additionally, we investigated if the expression of GLUT3 and GLUT5 sugar transporters was modulated by different glucose and fructose concentrations early after induction of chondrogenic differentiation. hBMSCs were seeded and cultured for 3 days in chondrogenic medium, composed of DMEM containing glucose at either 25 mM (HG), 5.5 mM (LG) or 1 mM (LLG), and 1% non-essential amino acids (Gibco), 1% ITS + (Corning), in the presence of 100 nM dexamethasone (Sigma-Aldrich), 50 µg/ml ascorbic acid-2 phosphate (Sigma-Aldrich) and 10 ng/ml TGF-β1 (Fitzgerald).

### RNA isolation and RT-qPCR

Total RNA was isolated from hBMSCs at day 0 (before cell seeding and chondrogenic commitment), day 3 and day 21 using TRI Reagent® (Molecular Research Centre Inc., Cincinnati, OH, USA) according to the manufacturer’s protocol. RNA quantity and quality were measured using the NanoDrop 1000 Spectrophotometer (Thermo Fisher). For reverse transcription (RT) of 1 µg of total RNA, TaqMan Reverse Transcription Kit (Applied Biosystems, Foster City, USA) was used. Quantitative PCR (qPCR) reactions were set up in 10 µl reaction mixtures containing TaqMan Gene Expression Master Mix (Thermo Fisher, Zürich, Switzerland), the appropriate set of primers and probes, DEPC-treated H_2_O and 10 ng of the cDNA template. The reaction program was set up as follows: 50 °C for 2 min, 95 °C for 10 min and 40 cycles of 95 °C for 15 s followed by an annealing/extension step at 60 °C for 1 min. All the qPCR runs were performed using QuantStudio 7 Flex Real-Time PCR System (Thermo Fisher). Technical replicates were used for each target gene and the different donors (biological replicates). The relative expression of genes *COL2A1*, *COL1A1*, *COL10A1*, *ACAN*, *RUNX2*, *SOX9*, *SP7*, *MMP13*, *PPARG, SLC2A3* and *SLC2A5* during chondrogenic differentiation were calculated as 2^(−Δ*Ct*)^, with *RPLP0* used as reference gene (see Additional file 1: Table S3 for assay details).

### Histology and Safranin-O staining

On day 21 samples were harvested and fixed in 70% methanol. One day before cutting, methanol was substituted with 5% sucrose and the samples were embedded in cryocompound (Thermo Fisher) for 15 min and then cryo-sectioned at a thickness of 10 µm. Safranin-O staining was then performed to evaluate the quality of cartilage matrix formation. The slides were washed in dH_2_O to remove the cryocompound, stained with Weigert’s Haematoxylin solution (Sigma-Aldrich) for 10 min and washed in tap water for 10 min. Sections were then stained for 6 min with Fast Green (Fluka #51275) and 15 min with Safranin-O (Sigma-Aldrich), followed by a wash with dH_2_O. After dehydration with increasing concentrations of ethanol, samples were transferred to xylene and coverslipped with Eukitt mounting medium (Sigma-Aldrich).

### Immunofluorescence (IF)

Cryosections were washed for 10 min with dH_2_O to remove the cryocompound, transferred to absolute methanol for 20 min and washed twice in 0.01% Tween 20 in PBS (PBS-T). Enzymatic treatment by 1 U/ml Hyaluronidase (Sigma-Aldrich, H3506) and 0.25 U/ml Chondroitinase ABC (Sigma-Aldrich, C2905) in PBS-T for 30 min at 37 °C allowed the matrix digestion for epitope retrieval. After washing in PBS-T, sections were transferred to blocking solution containing 5% horse serum (Vector laboratories, S-2000) in PBS-T for 30 min at room temperature,  Primary anti-type II collagen antibody (CIICI, see acknowledgement section) at a concentration of 4 μg/mL was added for 1 h at RT. Slides were washed with PBS, then the secondary antibody was added (Alexa Fluor 488 IgG 1:800) for 1 h at 37 °C. After washing with PBS, the nuclei were counterstained with 2-(4-Amidinophenyl)-1H-indole-6-carboxamidine (DAPI) 2.5 μg/mL and then coverslipped with Eukit mounting medium (Sigma-Aldrich, St. Louis, MO, USA).

hBMSCs were seeded on multi-well chamber slide and after the induction of chondrogenesis for 3 days they were fixed with methanol for 20 min and washed twice in 0.01% Tween 20 in PBS (PBS-T). After blocking with 3% BSA, samples were incubated with a GLUT3 (1:200 dilution) rabbit polyclonal antibody (#PA5-99169, Thermo Fisher) or a GLUT5 (1:50 dilution) mouse monoclonal antibody (#TA500575, Thermo Fisher) overnight at + 4 °C. After washing in PBS, samples were incubated with either a AF488-labelled secondary goat anti-rabbit antibody (1:800, #A-11008, Thermo Fisher) or a AF660-labelled secondary goat anti-mouse antibody (1:800, A-21055, Thermo Fisher) for 30 min at 4 °C. Proper unstained and secondary antibody-only samples were included for determining the fluorescence positivity regions. The nuclei were counterstained with 2-(4-Amidinophenyl)-1H-indole-6-carboxamidine (DAPI) at 2.5 μg/ml and then coverslipped with ProLong mounting solution (Thermo Fisher, P10144).

The fluorescent signal from type II collagen immunofluorescence was quantified in ImageJ (Fiji extension) [22]. In particular, the mean fluorescence intensity of each picture was calculated by applying an auto-thresholding and the filter "Despeckle" to reduce the noise around the main signal. Finally, the area of the main signal was selected, and the intensity of each pixel was measured and averaged. Additionally, the percentage of the area covered by the signal was calculated.

For the quantification of GLUT3 and GLUT5 IF signals, an auto threshold was applied and the filters “Fill holes” and “Dilate” were applied consequentially. Three different ROIs of each acquired image were selected and a mask was applied to select the area of the IF signal. The filter “Despeckle” was applied to reduce the noise. Additionally, for each ROI, the number of nuclei was calculated, and the IF signal of the nuclei was subtracted from the total signal.

### Analysis of glycosaminoglycan content by DMMB assay

After 21 days of culture, pellets were digested overnight with 0.5 mg/ml proteinase-K (Sigma-Aldrich) at 56 °C, followed by deactivation at 95 °C for 10 min. DNA content was quantified with Hoechst 33258 (Sigma-Aldrich) using a microplate reader (Victor3 Micro Plate Reader, PerkinElmer, Waltham, MA, USA) with excitation at 360 nm and emission at 465 nm according to published methodology [23]. In the same samples, quantification of glycosaminoglycans (GAG) was carried out via the 1, 9-Dimethyl-methylene Blue (DMMB) assay [24], with Chondroitin Sulphate from bovine trachea (Sigma-Aldrich) used as standard. Absorbance at 535 nm was measured.

### Statistical analysis

Statistical analysis was performed using GraphPad Prism 8 software (GraphPad Software, San Diego CA, USA). The results were analysed with a one-way ANOVA and Tukey's multiple comparison test.

## Results

### Effect of glucose and fructose on cartilage matrix deposition.

**Fig. 1:**
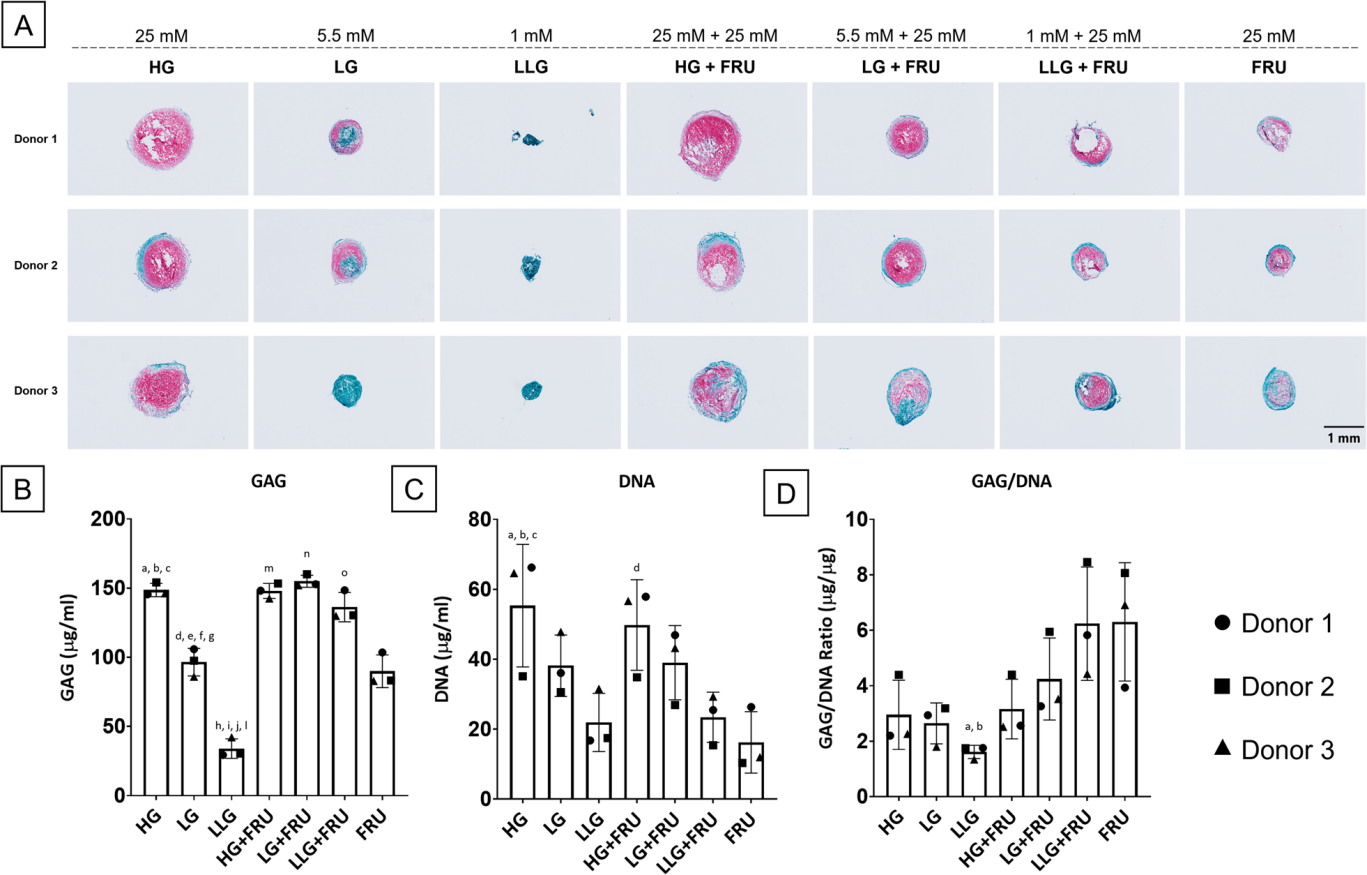
Influence of glucose and fructose on matrix deposition. Human BMSCs chondrogenic pellets were cultured for 21 days in the presence of different concentrations of glucose (25 mM, 5.5 mM and 1 mM), alone or in combination with 25 mM fructose. **A** Safranin-O/Fast Green staining on pellet cryosections, scale bar 1 mm. **B** Sulphated glycosaminoglycans content in pellets as determined by the DMMB assay. **C** DNA content in pellets. **D** Analysis of GAG production normalized to the DNA content (µg/µg). Data are expressed as mean ± SD (*n* = 3 BMSC donors), and the differences between each condition and the HG group were analysed with one-way ANOVA. a: *p* ≤ 0.0001; HG versus LG. b: *p* ≤ 0.0001; HG versus LLG. c: *p* ≤ 0.0001; HG versus FRU. d: *p* ≤ 0.0001. LG versus LLG. e: *p* ≤ 0.0001; LG versus HG + FRU. f: *p* ≤ 0.0001; LG versus LG + FRU. g: *p* ≤ 0.001; LG versus LLG + FRU. h: *p* ≤ 0.0001; LLG versus HG + FRU. i: *p* ≤ 0.0001; LLG versus LG + FRU. j: *p* ≤ 0.0001; LLG versus LLG + FRU. l: *p* ≤ 0.0001; LLG versus FRU. m: *p* ≤ 0.0001; HG + FRU versus FRU. n: *p* ≤ 0.0001; LG + FRU versus FRU. o: ≤ 0.001; LLG + FRU versus FRU. a: *p* ≤ 0.05; HG versus LLG; b: *p* ≤ 0.05; HG versus LLG + FRU. c: *p* ≤ 0.01; HG versus FRU. d: *p* ≤ 0.05; HG + FRU versus FRU. . a: *p* ≤ 0.05; LLG versus LLG + FRU b: *p *≤ 0.05; LLG versus FRU

After 21 days of differentiation in the presence of different concentrations of glucose or fructose, we investigated the quality of matrix deposition through histological evaluation and quantification of GAG content. The results in Fig. 1A show the effect of glucose depletion and fructose supplementation on matrix deposition. Glucose concentration was positively correlated with the size and intensity of Safranin-O staining. Supplementation of 25 mM fructose to a medium containing 1- or 5.5-mM glucose improved staining intensity and fructose alone also resulted in some GAG deposition. That observation was reflected analytically in both GAG (Fig. 1B) and DNA (Fig. 1C) quantification. Indeed, the content of DNA in pellets were influenced by glucose concentration in a dose-dependent manner. Moreover, except for the group with a high glucose concentration, fructose supplementation led to an increased GAG accumulation. This is also pointed out by the GAG/DNA quantification (Fig. 1D), that showed how fructose supplementation increased GAG production when added to 5.5 and 1 mM-glucose containing medium (groups LG + FRU and LLG + FRU, respectively), with the highest GAG/DNA values observed in the presence of fructose alone (FRU).

### Effect of glucose and fructose on type II collagen production.

**Fig. 2:**
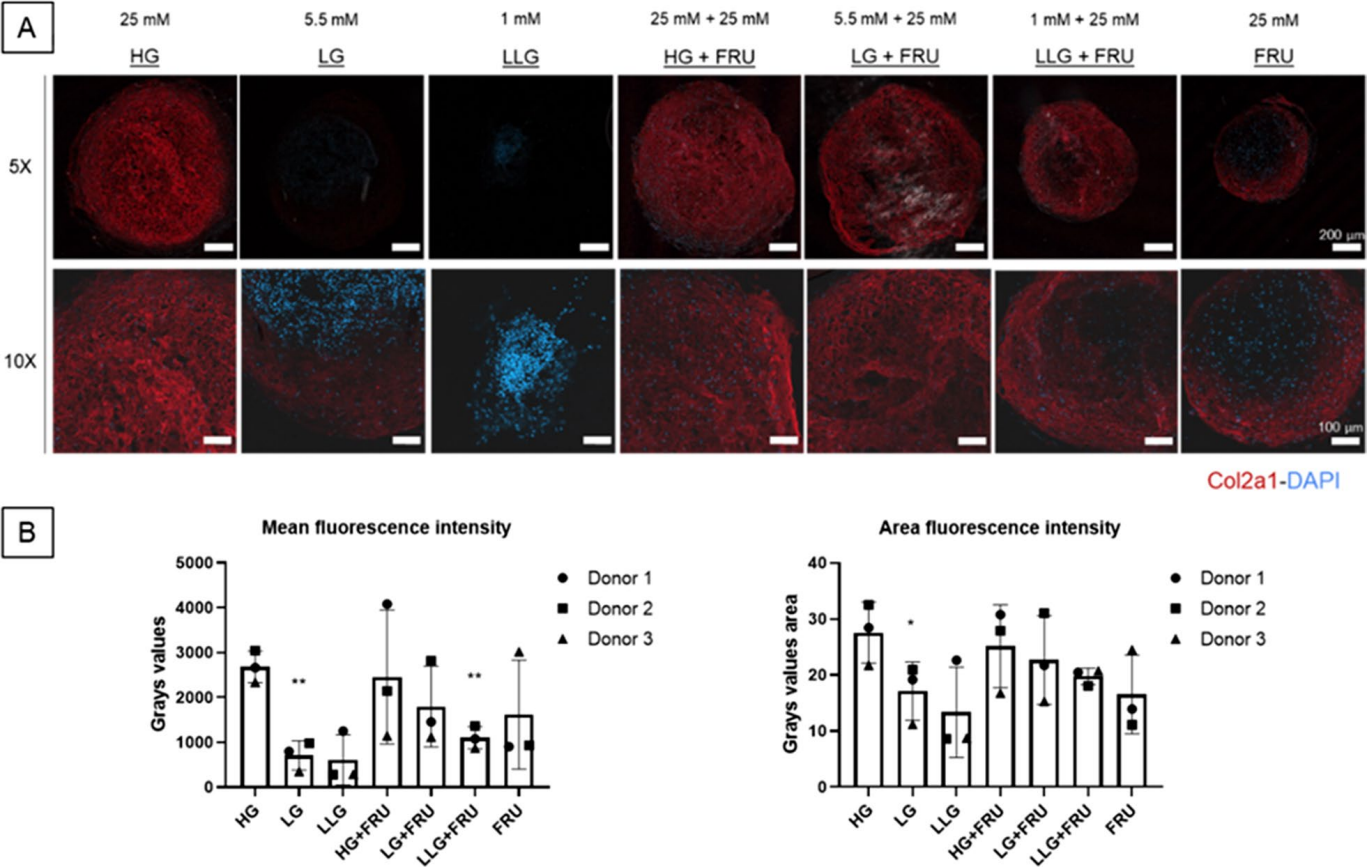
The chondrogenic potential of BMSCs in different cell culture media evaluated by type-II collagen immunostaining. **A** Representative pictures of *n* = 3 donors, here donor #2 is represented. Pellet cryosections stained for Collagen type II. The intensity of the fluorescent secondary antibody is directly proportional to the production of different collagen content, while DAPI (blue) stains cell nuclei. Scale bar (bottom right in each picture) = 200 µm (5× magnification) and 100 µm (10×). **B** Mean intensity and area of type-II collagen positive pellets. Data are expressed as mean ± SD (*p ≤ 0.05, **p ≤ 0.01 vs HG group). *N* = 3 BMSC donors

The protein expression of type II collagen was analysed to determine the influence of glucose and fructose on the quality of deposited matrix. Figure 2A shows representative images of type II collagen immunostaining. All donors expressed type II collagen after 21 days in culture in the standard medium containing 25 mM glucose (HG). Similarly to what was observed from Safranin-O/Fast Green staining, the decrease in glucose concentration limited significantly (LG **p ≤ 0.01) the production of type II collagen (Fig. 2B). When cells were supplemented with 25 mM fructose, type II collagen production was partially restored. Remarkably, the use of 25 mM fructose only also supported matrix deposition to a certain extent (Fig. 2B). In the LG + FRU group, pellets showed a similar production of matrix comparable to HG and HG + Fructose. This suggested that when using LG, the use of fructose can support type II collagen deposition with no significant change versus the HG group.

### Gene expression analysis of chondrogenic markers

**Fig. 3:**
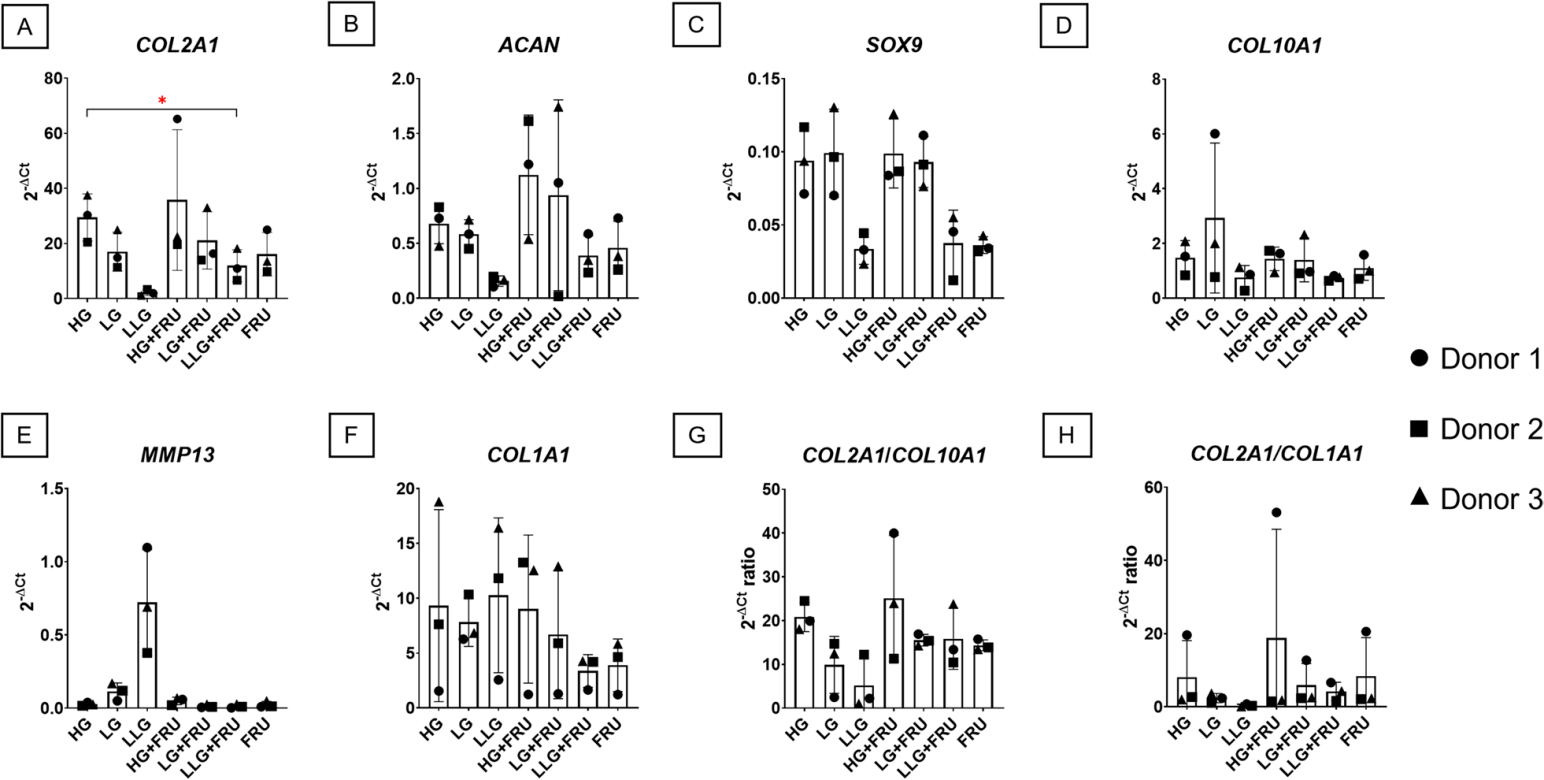
Influence of glucose and fructose on the expression of chondrogenic markers. Human BMSCs chondrogenic pellets were cultured for 21 days in presence of different concentrations of glucose (25 mM, 5.5 mM and 1 mM), alone or in combination of 25 mM fructose. Data are expressed as mean ± SD; significant difference from the HG group is marked by a red asterisk (*p ≤ 0.05). *n* = 3 BMSC donors

We further evaluated the effect of different concentrations of glucose on chondrogenic commitment after 21 days by gene expression analysis. For most of the genes, there were no significant differences between the groups, even though some trends could be observed. In fact, a higher concentration of glucose generally corresponded to an increased expression of all the chondrogenic markers. Vice versa, a lower concentration of glucose enhanced the expression of *MMP13*. The presence of fructose alone did not fully support the expression of the chondrogenic markers but, at the same time, it did not enhance the expression of hypertrophic markers. In accordance with the histological results, the expression of *COL2A1* (Fig. 3A) was correlated with glucose concentrations; the use of 1 mM glucose indeed resulted in the lowest levels of this gene. On the other hand, the use of 25 mM fructose alone resulted in *COL2A1* levels similar to the LG group, and it slightly improved *COL2A1* expression when supplemented to glucose containing medium. However, the combination of fructose with 1 mM glucose did not lead to the same levels of *COL2A1* production as the standard 25 mM glucose concentration alone (*p* ≤ 0.05).

The expression of *ACAN* and *SOX9* (Fig. 3B, C) did not show any significant differences among the groups, though there was a tendency towards higher gene expression levels with higher glucose concentration.

Among hypertrophy markers, *COL10A1* expression (Fig. 3D) was comparable among groups. Even though not statistically significant, *MMP13* (Fig. 3E) showed higher expression levels in the LLG group, and the addition of fructose was completely counteracting this effect.

Expression of *COL1A1* showed a tendency towards lower levels in presence of fructose (Fig. 3F). To better evaluate the chondrogenic potential of pellets, the ratios between the expression of *COL2A1* and *COL10A1* (Fig. 3G), or *COL2A1* and *COL1A1* (Fig. 3H) were calculated. The analysis showed a trend towards a correlation of the ratios to the glucose concentration, seemingly with no effect of fructose supplementation.

### Gene expression analysis of osteogenic and adipogenic markers

**Fig. 4:**
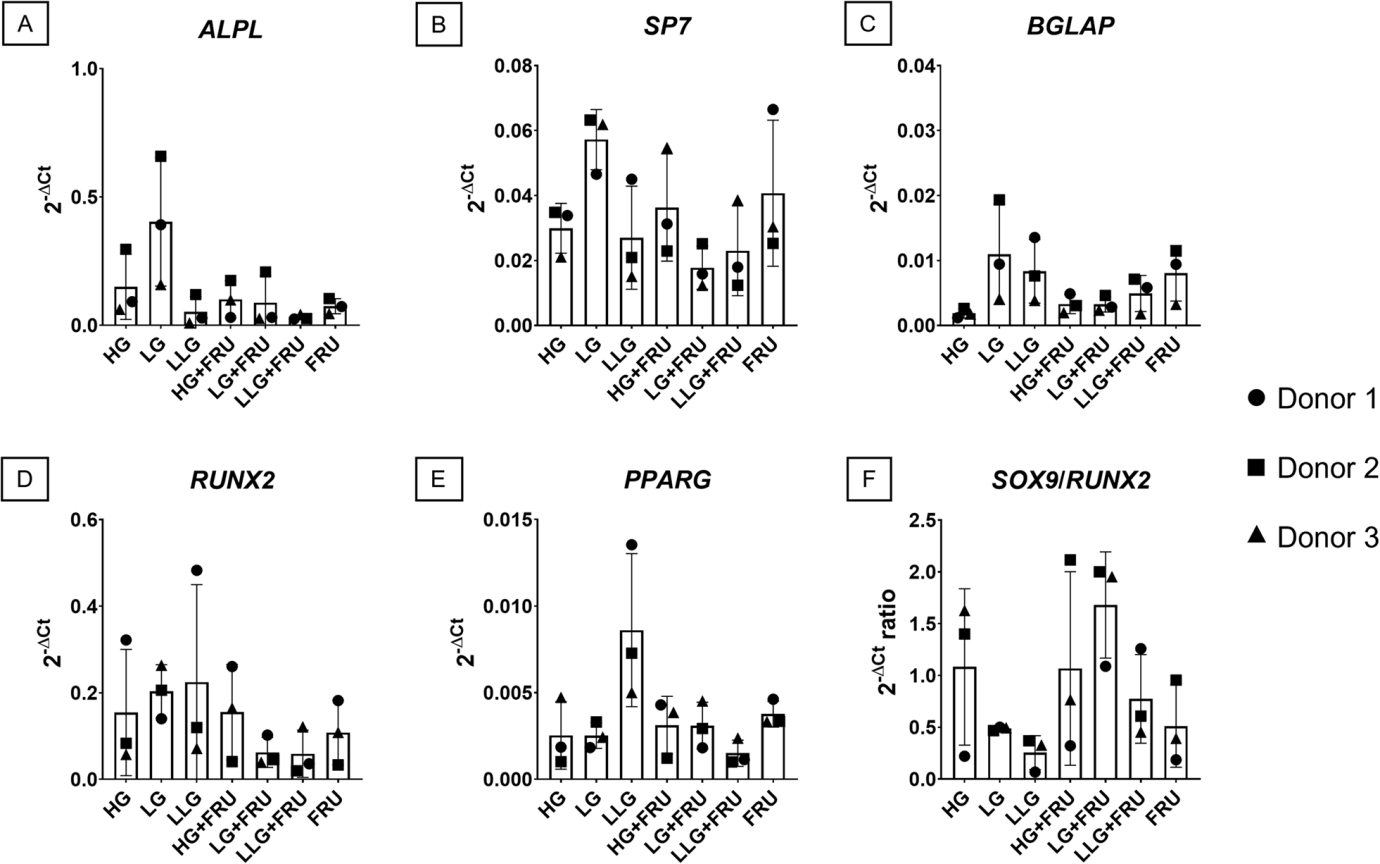
Influence of glucose and fructose on the expression of osteogenic and adipogenic markers. Human BMSCs chondrogenic pellets were cultured for 21 days in presence of different concentrations of glucose (25 mM, 5.5 mM and 1 mM), alone or in the presence of 25 mM of fructose. Data are expressed as mean ± SD. N = 3 BMSC donors

We additionally evaluated the effect of glucose and fructose on the expression of markers associated with endochondral ossification and adipogenic differentiation, because we were interested to observe possible non-specific regulation of genes associated to alternative differentiation pathways that could interfere with a stable chondrogenesis. The expression of *ALPL*, *SP7*, *BGLAP,* and *RUNX2* (Fig. 4A–D) showed similar trends, where the addition of fructose did not promote their expression. The non-statistically different increase in *PPARG* levels in the LLG group seemed to be counteracted in the presence of fructose. Different concentrations of glucose and fructose seemed to influence the balance between the chondrogenic and the osteogenic fate. Indeed, at both day 3 (Additional file 1: Fig. S1) and day 21 (Fig. 4F), fructose supplementation to a medium containing 5.5 or 1 mM glucose tended to increase the *SOX9*/*RUNX2,* indicative of a more stable chondrogenic phenotype.

### Expression of glucose and fructose transporters GLUT3 and GLUT5

**Fig. 5:**
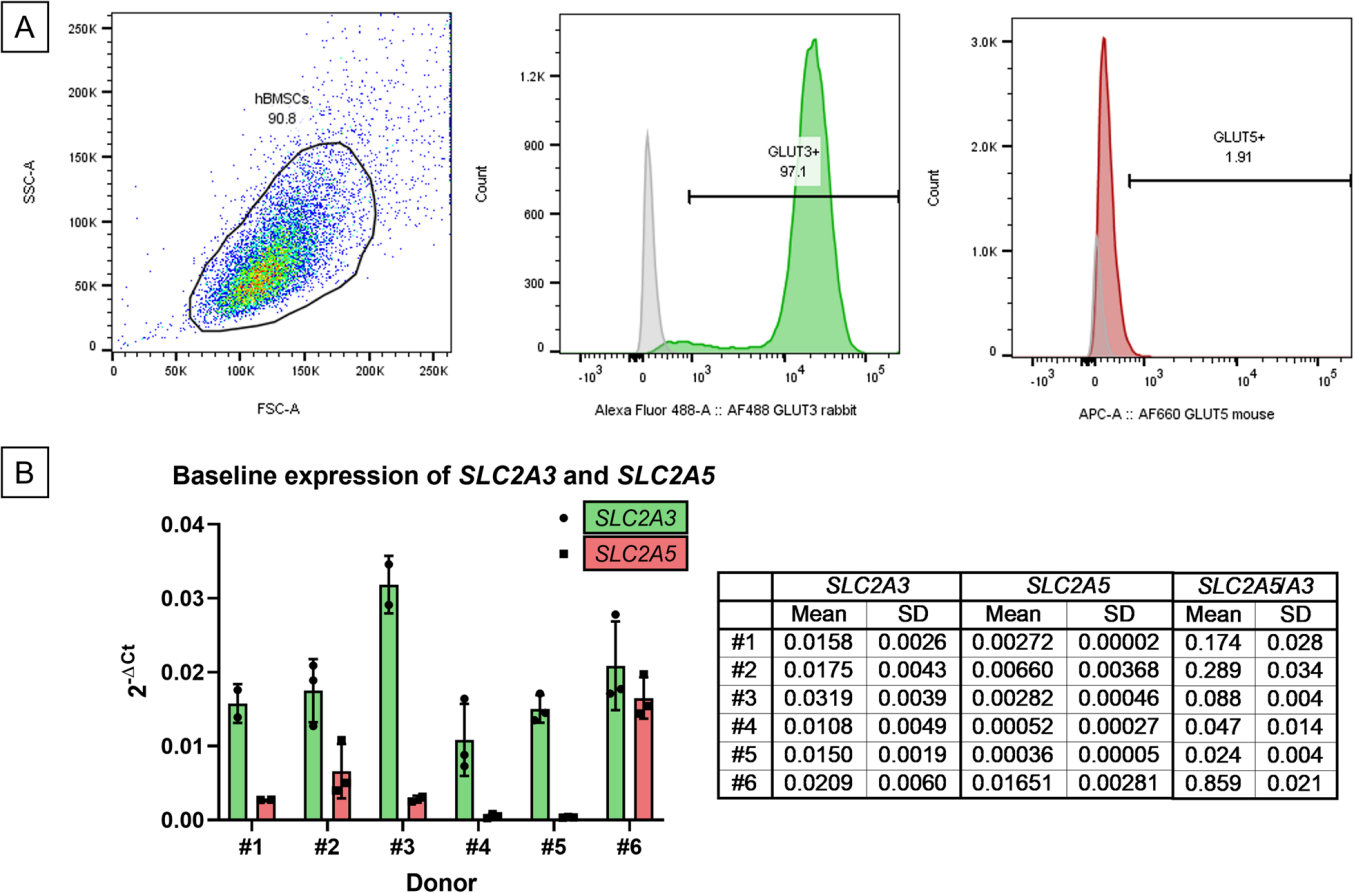
Analysis of GLUT3 and GLUT5 on naïve hBMSCs. **A** Representative image depicting flow cytometry analysis of GLUT3 and GLUT5 expression on naïve hBMSCs. The gating strategy included the first selection of cells based on the FSC/SSC dot-plot. After the selection of the hBMSCs population, the range of GLUT3 (green histogram) and GLUT5 (red) positivity was set based on the negative controls (staining with secondary antibody only, grey peaks in histogram overlays). **B** Analysis of GLUT3 (SLC2A3) and GLUT5 (SLC2A5) expression by qPCR. The graph represents the gene expression levels of the single donors performed in three technical replicates, while the table summarizes the expression ratios between the two genes (2^−Δ*Ct*^ ratio)

Flow cytometry allowed the evaluation of the expression of two sugar transporters, GLUT3 and GLUT5, on naïve (monolayer-expanded) hBMSCs. A representative depiction of flow cytometry gating strategy and results is shown in Fig. 5A. hBMSCs showed a high expression of GLUT3, with GLUT3 + cells accounting for 98.4 ± 1.27% of events gated in the hBMSC population. The mean fluorescence intensity was 23,222 ± 2466. On the contrary, GLUT5 was expressed only by a small proportion of cells (3.04 ± 1.23% of events gated in the hBMSC population), with a mean fluorescence intensity of 1224 ± 296. The full results for GLUT3 and GLUT5 expression levels in hBMSCs are reported in Additional file 1: Table S4.

Naïve hBMSCs from the same donors were also tested for *SLC2A3* and *SLC2A5* gene expression by qPCR (Fig. 5B). The results showed that the baseline expression of *SLC2A3* was rather stable in naïve BMSCs among donors (2^−Δ*Ct*^: 0.0171 ± 0.0079, normalized to *RPLP0* expression). On the contrary, the expression of *SLC2A5* was more variable, ranging from a relative level of 0.0003 to 0.0149 (average 0.0043 ± 0.0054; normalized to *RPLP0*). The expression of *SLC2A5* was generally lower than *SLC2A3*, as confirmed by flow cytometry, although their relative proportion was different among donors (table embedded in Fig. 5B).

**Fig. 6:**
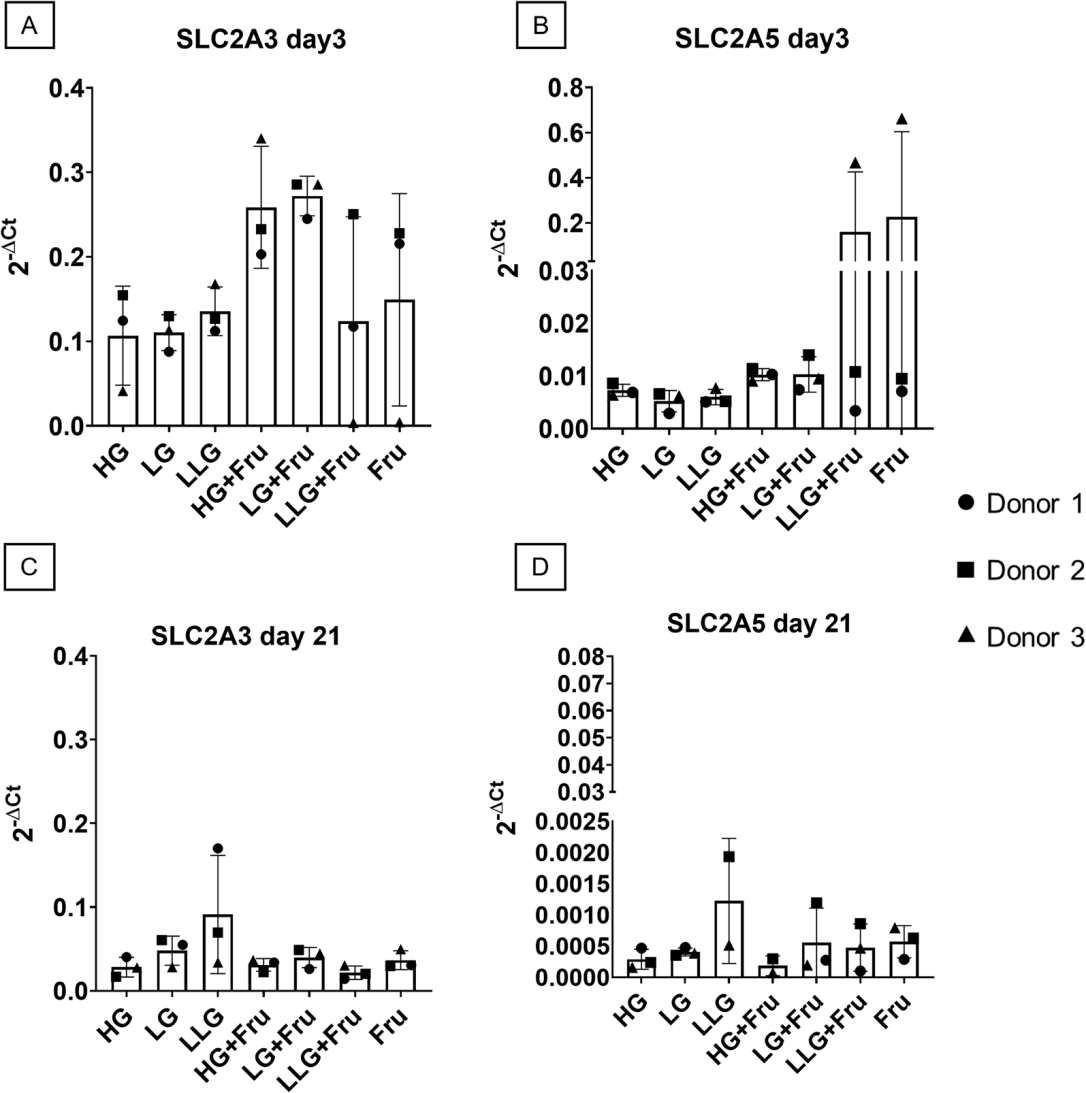
Influence of glucose and fructose on *SLC2A3* and *SLC2A5* gene expression during BMSC chondrogenesis. Cells were cultured for 3 or 21 days in the presence of different concentrations of glucose (25 mM, 5.5 mM and 1 mM), alone or in combination with 25 mM fructose. **A** *SLC2A3* and **B** *SLC2A5* expression after 3 days of chondrogenic commitment. **C** *SLC2A3* and **D** *SLC2A5* levels after 21 days of differentiation

Additionally, we investigated if the expression of GLUT3 and GLUT5 sugar transporters was modulated by different glucose and fructose concentrations after induction of chondrogenic differentiation. The results showed no significant differences in the expression of *SLC2A3* and *SLC2A5* at day 3 (Fig. 6A, B). However, a trend for a higher expression of *SLC2A5* was observed in the LLG + Fru group or when the medium was supplemented with fructose only. Interestingly, after 21 days of differentiation, both the levels of *SLC2A3* and *SLC2A5* transcripts were lower compared to day 3. We found no statistically significant differences among groups treated with different concentration of sugars, with a trend towards an upregulation of the transporters with lower concentrations of glucose in the medium (Fig. 6C, D).

**Fig. 7:**
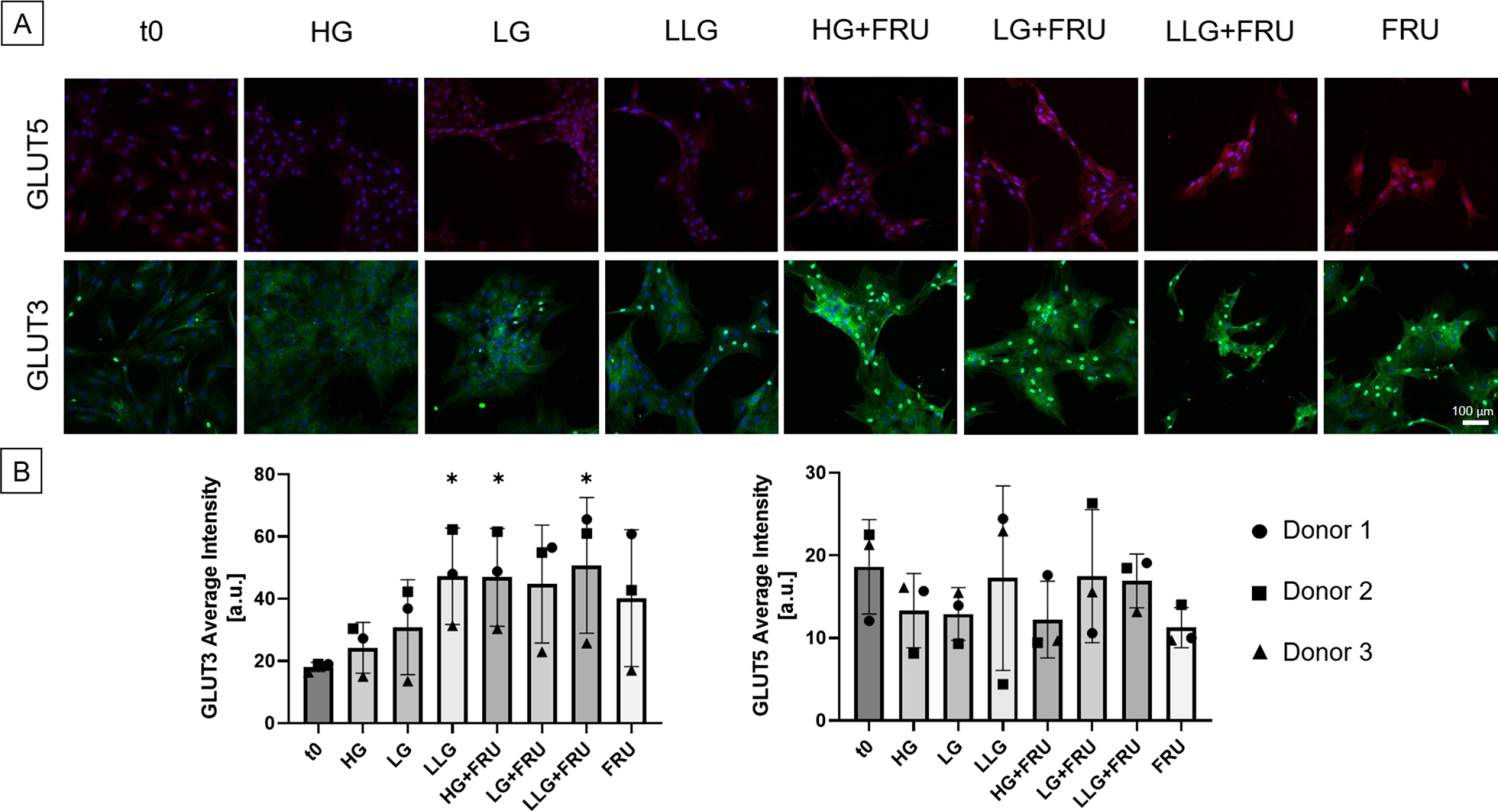
Qualitative evaluation of GLUT3 and GLUT5 protein expression during early chondrogenic commitment by immunostaining. **A** Representative pictures of *n* = 3 donors, here donor #2 is represented. BMSCs, cultured in monolayer and in the presence of chondrogenic medium, were stained against GLUT5 and GLUT3. The intensity of fluorescent secondary antibody is directly proportional to the production of GLUT5 and GLUT3, while DAPI (blue) stains cell nuclei. Scale bar (bottom right picture) = 100 µm (10×). **B** Mean intensity and area of GLUT3 and GLUT5 positive cells. Data are expressed as mean ± SD (*p ≤ 0.05, **p ≤ 0.01 vs HG group). *N* = 3 BMSC donors

In terms of protein levels, the lowest IF signal intensity for GLUT3 was found in the control group t0 (Fig. 7). At day 3, GLUT3 expression increased with a decrease in the glucose concentration from 25 (HG) to 1 mM (LLG). The groups that showed the highest expression of GLUT3 were the fructose-supplemented groups that also showed a higher nuclear localization. GLUT5, as expected, was expressed on the cellular membrane especially in the fructose-supplemented groups and we did not observe any nuclear localization. However, we did not find any statistical differences in terms of GLUT5 expression between the fructose-supplemented group and the fructose-free counterparts.

## Discussion

This study showed the different impact of the use of glucose and fructose during chondrogenic differentiation of human BMSCs. For the first time, fructose has been used as a metabolic substrate to check how it affects chondrogenesis in the absence or with low levels of glucose. The results indicate that low concentrations of glucose (5.5 and 1 mM) are not sufficient to induce a good chondrogenic differentiation and cartilage matrix deposition. In the presence of very low glucose concentrations (1 mM), BMSCs were not able to undergo chondrogenic differentiation, as demonstrated by histology and gene expression analysis. It has been previously reported that implanted hBMSCs consume their glycolytic reserves through glycolysis in less than 24 h, leading to cell death within 3 days post-implantation [25]. However, this scenario changes when fructose is supplemented. Indeed, fructose supplementation enhanced matrix production when added to a medium containing 5.5 or 1 mM of glucose. This suggests that fructose can be also used by BMSCs as an alternative energy source for matrix formation. Indeed, glucose is an intermediate in the biosynthesis of GAG [12]. Fructose alone could also support a low level of GAG production, as demonstrated by histology and biochemical evaluations.

Fructose alone was not able to sustain high expression levels of chondrogenic markers, if compared with glucose alone. This indicates that glucose is the primary energy source of BMSCs during differentiation. However, fructose presence could counteract the high expression of *MMP13*, which was observed with the lowest glucose concentrations. Fructose, furthermore, did not influence significantly the expression of markers related to cartilage hypertrophy, endochondral ossification, or adipogenesis, and its supplementation to low glucose concentrations tended to lower the expression of such markers, suggesting that fructose can help maintaining a stable chondrogenic differentiation under limited glucose conditions. The analysis of type II collagen expression supports this hypothesis, as its protein levels could be improved when adding fructose to low glucose concentrations.

To confirm the uptake of fructose, the expression of two sugar transporters was investigated. GLUT3 (*SLC2A3*) is a high-affinity transporter for glucose (Km ~ 1.8 mM), whereas GLUT5 (*SLC2A5*) is responsible for the uptake of fructose (Km ~ 8.3 mM) together with GLUT2 [26]. GLUT3 is also involved in the transport of mannose, galactose, and xylose but is unable to transport fructose and has been shown to be present in all cell types. GLUT3 can also carry dehydroascorbic acid [13], which participates in collagen synthesis [27]. Together with GLUT1, GLUT3 is ubiquitously distributed on the cell membrane, suggesting the important role in cell survival during basal glucose uptake [28]. On the contrary, GLUT5 is mainly expressed in small intestine, kidney, adipocytes, skeletal muscle, and brain but it has not been described yet in bone marrow stem cells [13, 17].

While GLUT3 seemed to be expressed homogeneously at high levels in naïve hBMSCs, GLUT5 expression was more variable and lower than GLUT3. Interestingly, after 3 days of chondrogenic commitment, in one donor GLUT5 was upregulated in the presence of fructose alone or in combination with 1 mM glucose, indicating that a feedback mechanism might exist to increase fructose uptake in case of inadequate supply of glucose and in the presence of fructose. In groups with both glucose and fructose, GLUT3 was expressed at higher levels, suggesting that the use of fructose may positively modulate the expression of GLUT3 and its high localization at the nuclei. This mechanism has been not observed previously in BMSC cells. Weisowa et al. [29] described the translocation of GLUT3 on plasma membrane in neurons after glutamate excitation, showing that this event occurs for increased glucose uptake in response to the energy deprivation associated with glutamate receptor overactivation.

From a clinical perspective, it is important to maintain an optimal environment for MSC differentiation, e.g. in microfracture surgery for cartilage repair. The physiological glucose concentration in knee joints is similar to that in the blood level, which is between 3 and 5 mM or 90–100% of blood glucose in healthy individuals or patients with non-inflammatory changes [7]. However, after an arthroscopic procedure, which is usually utilized to perform microfracturing, the synovial fluid is washed out of the joint and the lavage solution is left. Studies have shown that both saline and Ringer lactate, which do not contain any glucose or fructose, can be detrimental to chondrocyte health if left in contact with the cartilage, and cartilage metabolism appears to be damaged for up to 2 weeks [30]. A better control on the environment to which the blood clot is exposed during the first phases of repair might be beneficial to improve cell differentiation potential and this control might include a balanced supplementation of sugars to support proper cell metabolism.

### Study strengths and limitations

This study has both strengths and limitations. To the best of our knowledge, this study is the first providing evidence on the expression of fructose transporters on human BMSCs and of its effects during chondrogenic differentiation in vitro, giving insights on how cells respond to different sugar composition and levels.

One of the limitations is that this study has been performed in vitro only, with no further in vivo or clinical evidence. Moreover, intra-donor variability is a common issue when using primary human cells, and we have no access to clinical information about the cell donors, including pre-existing pathologies. Being able to correlate in vitro results to the presence of certain metabolic diseases, such as diabetes or metabolic syndrome, would increase the translational relevance of the results.

Other limitations can be identified in the in vitro model. The present study focused on day 21 chondrogenic differentiation of BMSCs. Shorter or longer time-points would also allow to understand more in details the effects of sugars on cell differentiation mechanisms.

The use of 25 mM fructose is also supraphysiological, but we chose this concentration to have a direct comparison with the levels of glucose commonly used in chondrogenic differentiation in vitro.

Further experiments need to be performed in order to understand what the mechanism behind glucose and fructose transporter regulation is and how this ultimately affect cell fate and metabolism.

## Conclusions

The depletion of glucose during chondrogenic commitment reduced the yield of differentiation, however, the use of fructose in a low glucose environment positively drove cell commitment. Supplementation of cell culture medium with alternative energetic substrates like fructose can therefore support cell commitment and energy metabolism.

This study contributes to a clearer understanding of sugar utilization during chondrogenic differentiation. This work demonstrates, for the first time, the presence of the fructose transporter GLUT5 in human BMSC and how it is modulated during chondrogenic commitment. Additionally, we demonstrated that the use of fructose alone or in combination with limited amount of glucose does not inhibit differentiation, rather it supports differentiation in a situation with a limited supply of the main energy substrate. Additional research is needed to establish if lower concentration of fructose alone or in combination with low and physiological concentration of glucose can explored for its potentiality to maintain a stable differentiation of BMSCs into chondrocytes. In our future studies, we will focus on understanding the importance of glucose and fructose concentration in vivo to foster the translational potential of our findings to improve cartilage repair strategies.

## Supplementary Information

**Additional file 1**. Supplementary information including cell donor information, medium composition, details of assays used for gene expression analysis, summary of GLUT3 and GLUT5 flow cytometry analysis, analysis of SOX9/RUNX2 ratio.

## Data Availability

All data generated or analysed during this study are available from the corresponding author on reasonable request.

## References

[1] Russell AL, Lefavor R, Durand N, Glover L, Zubair AC (2018). Modifiers of mesenchymal stem cell quantity and quality. Transfusion.

[2] Wang M, Yuan Q, Xie L (2018). Mesenchymal stem cell-based immunomodulation: properties and clinical application. Stem Cells Int.

[3] Jo CH, Lee YG, Shin WH, Kim H, Chai JW, Jeong EC (2014). Intra-articular injection of mesenchymal stem cells for the treatment of osteoarthritis of the knee: a proof-of-concept clinical trial. Stem cells.

[4] Zuncheddu D, Della Bella E, Schwab A, Petta D, Rocchitta G, Generelli S (2021). Quality control methods in musculoskeletal tissue engineering: from imaging to biosensors. Bone Res.

[5] Basoli V, Della Bella E, Kubosch EJ, Alini M, Stoddart MJ (2021). Effect of expansion media and fibronectin coating on growth and chondrogenic differentiation of human bone marrow-derived mesenchymal stromal cells. Sci Rep.

[6] Kovermann NJ, Basoli V, Della Bella E, Alini M, Lischer C, Schmal H (2019). BMP2 and TGF-beta cooperate differently during synovial-derived stem-cell chondrogenesis in a dexamethasone-dependent manner. Cells.

[7] Mantripragada VP, Kaplevatsky R, Bova WA, Boehm C, Obuchowski NA, Midura RJ (2021). Influence of glucose concentration on colony-forming efficiency and biological performance of primary human tissue-derived progenitor cells. Cartilage.

[8] Zhou S, Cui Z, Urban JP (2004). Factors influencing the oxygen concentration gradient from the synovial surface of articular cartilage to the cartilage–bone interface: a modeling study. Arthritis Rheum.

[9] Otte P (1991). Basic cell metabolism of articular cartilage. Manometric Stud Z Rheumatol.

[10] Heywood HK, Nalesso G, Lee DA, Dell'accio F (2014). Culture expansion in low-glucose conditions preserves chondrocyte differentiation and enhances their subsequent capacity to form cartilage tissue in three-dimensional culture. Biores Open Access.

[11] Windhaber RA, Wilkins RJ, Meredith D (2003). Functional characterisation of glucose transport in bovine articular chondrocytes. Pflugers Arch.

[12] Hollander JM, Zeng L (2019). The emerging role of glucose metabolism in cartilage development. Curr Osteoporos Rep.

[13] Mobasheri A, Vannucci SJ, Bondy CA, Carter SD, Innes JF, Arteaga MF (2002). Glucose transport and metabolism in chondrocytes: a key to understanding chondrogenesis, skeletal development and cartilage degradation in osteoarthritis. Histol Histopathol.

[14] Sun C, Lan W, Li B, Zuo R, Xing H, Liu M (2019). Glucose regulates tissue-specific chondro-osteogenic differentiation of human cartilage endplate stem cells via O-GlcNAcylation of Sox9 and Runx2. Stem Cell Res Ther.

[15] Theytaz F, De Giorgi S, Hodson L, Stefanoni N, Rey V, Schneiter P (2014). Metabolic fate of fructose ingested with and without glucose in a mixed meal. Nutrients.

[16] Herman MA, Samuel VT (2016). The sweet path to metabolic demise: fructose and lipid synthesis. Trends Endocrinol Metab.

[17] Burant CF, Bell GI (1992). Mammalian facilitative glucose transporters: evidence for similar substrate recognition sites in functionally monomeric proteins. Biochemistry.

[18] Elger BS, Caplan AL (2006). Consent and anonymization in research involving biobanks: differing terms and norms present serious barriers to an international framework. EMBO Rep.

[19] der Medizinischen Wissenschaften SA. Biobanken: Gewinnung, Aufbewahrung und Nutzung von menschlichem biologischem Material für Ausbildung und Forschung. Medizinisch-ethische Richtlinien und Empfehlungen Basel, Switzerland: Schweizer Akademie der Medizinischen Wissenschaften [Google Scholar]. 2006.

[20] Gardner OF, Alini M, Stoddart MJ (2015). Mesenchymal stem cells derived from human bone marrow. Methods Mol Biol.

[21] Johnstone B, Hering TM, Caplan AI, Goldberg VM, Yoo JU (1998). In vitrochondrogenesis of bone marrow-derived mesenchymal progenitor cells. Exp Cell Res.

[22] Schneider CA, Rasband WS, Eliceiri KW (2012). NIH Image to ImageJ: 25 years of image analysis. Nat Methods.

[23] Labarca C, Paigen K (1980). A simple, rapid, and sensitive DNA assay procedure. Anal Biochem.

[24] Farndale RW, Buttle DJ, Barrett AJ (1986). Improved quantitation and discrimination of sulphated glycosaminoglycans by use of dimethylmethylene blue. Biochem Biophys Acta.

[25] Moya A, Paquet J, Deschepper M, Larochette N, Oudina K, Denoeud C (2018). Human mesenchymal stem cell failure to adapt to glucose shortage and rapidly use intracellular energy reserves through glycolysis explains poor cell survival after implantation. Stem Cells.

[26] Thorens B, Mueckler M (2010). Glucose transporters in the 21st century. Am J Physiol Endocrinol Metab.

[27] Shikhman AR, Brinson DC, Lotz MK (2004). Distinct pathways regulate facilitated glucose transport in human articular chondrocytes during anabolic and catabolic responses. Am J Physiol Endocrinol Metab.

[28] Burant C, Takeda J, Brot-Laroche E, Bell G, Davidson N (1992). Fructose transporter in human spermatozoa and small intestine is GLUT5. J Biol Chem.

[29] Weisová P, Concannon CG, Devocelle M, Prehn JH, Ward MW (2009). Regulation of glucose transporter 3 surface expression by the AMP-activated protein kinase mediates tolerance to glutamate excitation in neurons. J Neurosci.

[30] Mathies B (2006). Effects of viscoseal, a synovial fluid substitute, on recovery after arthroscopic partial meniscectomy and joint lavage. Knee Surg Sports Traumatol Arthrosc.

