# Supplementary material for: Effect of glucose depletion and fructose administration during chondrogenic commitment in human bone marrow-derived stem cells
Source: Stem Cell Res Ther. 2022 Dec 27;13:533. doi: 10.1186/s13287-022-03214-2 (PMC9795608; doi:10.1186/s13287-022-03214-2)
Supplement: Supplementary file 1 — Additional file 1. Supplementary information including cell donor information, medium composition, details of assays used for gene expression analysis, summary of GLUT3 and GLUT5 flow cytometry analysis, analysis of SOX9/RUNX2 ratio. [file 13287_2022_3214_MOESM1_ESM.docx]

Supplementary materials

**Table S1.** Donor information

| **Donor #** | **Sex** | **Age** |
| --- | --- | --- |
| **1** | Female | 48 |
| **2** | Female | 83 |
| **3** | Male | 73 |
| **4** | Female | 79 |
| **5** | Female | 87 |
| **6** | Male | 76 |

**Table S2.** Differences in medium composition for each group. HG = 25 mM, LG = 5.5 mM, LLG=1 mM.

| Group  name | Glucose concentration | Fructose concentration |
| --- | --- | --- |
| HG | 25 mM / 4.5 g/L | - |
| LG | 5.5 mM / 1 g/L | - |
| LLG | 1 mM / 0.18 g/L | - |
| HG+Fru | 25 mM / 4.5 g/L | 25 mM / 4.5 g/L |
| LG+Fru | 5.5 mM / 1g /L | 25 mM / 4.5 g/L |
| LLG+Fru | 1 mM | 25 mM / 4.5 g/L |
| Fru | - | 25 mM / 4.5 g/L |

**Table S3.** List and details of primers and probes used for gene expression analysis

| **Gene** | **Assay Details** |
| --- | --- |
| *ACAN* | Forward primer: 5'-AGTCCTCAAGCCTCCTGTACTCA-3'  Reverse primer: 5'-CGGGAAGTGGCGGTAACA-3'  Probe: 5'-CCGGAATGGAAACGTGAATCAGAATCAACT-3' |
| *ALPL* | TaqMan Gene expression assay Hs00758162_m1 |
| *BGLAP* | Forward primer: 5'-AAGAGACCCAGGCGCTACCT-3'  Reverse primer: 5'-AACTCGTCACAGTCCGGATTG-3'  Probe: 5'-ATGGCTGGGAGCCCCAGTCCC-3' |
| *COL1A1* | Forward primer: 5'-CCCTGGAAAGAATGGAGATGAT-3'  Reverse primer: 5'-ACTGAAACCTCTGTGTCCCTTCA-3'  Probe: 5'-CGGGCAATCCTCGAGCACCCT-3' |
| *COL2A1* | Forward primer: 5'-GGCAATAGCAGGTTCACGTACA-3'  Reverse primer: 5'-GATAACAGTCTTGCCCCACTTACC-3'  Probe: 5'-CCTGAAGGATGGCTGCACGAAACATAC-3' |
| *COL10A1* | Forward primer: 5'-ACGCTGAACGATACCAAATG-3'  Reverse primer: 5'-TGCTATACCTTTACTCTTTATGGTGTA-3'  Probe: 5'-ACTACCCAACACCAAGACACAGTTCTTCATTCC-3' |
| *MMP13* | Forward primer: 5'-CGGCCACTCCTTAGGTCTTG-3'  Reverse primer: 5'-TTTTGCCGGTGTAGGTGTAGATAG-3'  Probe: 5'-CTCCAAGGACCCTGGAGCACTCATGT-3' |
| *PPARG* | TaqMan Gene expression assay Hs00234592_m1 |
| *RPLP0* | Forward primer: 5'-TGGGCAAGAACACCATGATG-3'  Reverse primer: 5'-CGGATATGAGGCAGCAGTTTC-3'  Probe: 5'-AGGGCACCTGGAAAACAACCCAGC-3' |
| *RUNX2* | Forward primer: 5'-AGCAAGGTTCAACGATCTGAGAT-3'  Reverse primer: 5'-TTTGTGAAGACGGTTATGGTCAA-3'  Probe: 5'-TGAAACTCTTGCCTCGTCCACTCCG-3' |
| *SLC2A3* | TaqMan Gene expression assay Hs00359840_m1 |
| *SLC2A5* | TaqMan Gene expression assay Hs01086390_m1 |
| *SOX9* | TaqMan Gene expression assay Hs00165814_m1 |
| *SP7* | Forward primer: 5'-CCTGCTTGAGGAGGAAGTTCA-3'  Reverse primer: 5'-GGCTAGAGCCACCAAATTTGC-3'  Probe: 5'-TCCCCTGGCCATGCTGACGG-3' |

**Table S4.** Summary of results of GLUT3 and GLUT5 staining. N=4 donors. The table reports the percentage of positive cells contained in the hBMSCs population (see Figure 1A in the main text), their mean fluorescence intensity, and the standard deviation (SD) of fluorescence in the positive population. The average results of 4 donors (last row) are reported as mean ± standard deviation.

| GLUT3 staining | | | |
| --- | --- | --- | --- |
| Donor # | Freq. of Parent (%) | Mean Fluorescence Intensity | Fluorescence SD |
| 2 | 97.1 | 22338 | 13505 |
| 4 | 97.6 | 21253 | 12224 |
| 5 | 99.7 | 26829 | 14211 |
| 6 | 99.3 | 22466 | 11597 |
| Average  (n=4 donors) | **98.4 ± 1.27** | **23222 ± 2466** | **12884 ± 1189** |

| GLUT5 staining | | | |
| --- | --- | --- | --- |
| Donor # | Freq. of Parent (%) | Mean Fluorescence Intensity | Fluorescence SD |
| 2 | 1.91 | 1498 | 9584 |
| 4 | 2.52 | 1418 | 10661 |
| 5 | 4.76 | 847 | 639 |
| 6 | 2.98 | 1132 | 7344 |
| Average  (n=4 donors) | **3.04 ± 1.23** | **1224 ± 296** | **7057 ± 4496** |

Supplementary Figure 1. **Influence of glucose and fructose on SOX9/RUNX2 ratio at early BMSC chondrogenesis.** Cells were cultured for 3 days in presence of different concentrations of glucose (25 mM, 5.5 mM and 1 mM), alone or in combination with 25 mM fructose. Data are expressed as mean ± SD; significant difference from the HG group is marked by red asterisks (* p ≤0.05, ** p ≤0.01).
